# Improved measurement for mothers, newborns and children in the era of the Sustainable Development Goals

**DOI:** 10.7189/jogh.06.010506

**Published:** 2016-06

**Authors:** Tanya Marchant, Jennifer Bryce, Cesar Victora, Allisyn C Moran, Mariam Claeson, Jennifer Requejo, Agbessi Amouzou, Neff Walker, Ties Boerma, John Grove

**Affiliations:** 1 Department of Disease Control, London School of Hygiene and Tropical Medicine, London, UK; 2 Department of International Health, Johns Hopkins Bloomberg School of Public Health, Baltimore, USA; 3 International Center for Equity in Health, Post–Graduate Programme in Epidemiology, Federal University of Pelotas, Pelotas, Brazil; 4 Global Health Fellows Program II, Bureau for Global Health, US Agency for International Development, Washington, USA; 5 Bill & Melinda Gates Foundation, Seattle, WA, USA; 6 UNICEF, New York, NY, USA; 7 WHO, Health Systems and Innovation, Geneva, Switzerland

## Abstract

**Background:**

An urgent priority in maternal, newborn and child health is to accelerate the scale–up of cost–effective essential interventions, especially during labor, the immediate postnatal period and for the treatment of serious infectious diseases and acute malnutrition.  Tracking intervention coverage is a key activity to support scale–up and in this paper we examine priorities in coverage measurement, distinguishing between essential interventions that can be measured now and those that require methodological development.

**Methods:**

We conceptualized a typology of indicators related to intervention coverage that distinguishes access to care from receipt of an intervention by the population in need.  We then built on documented evidence on coverage measurement to determine the status of indicators for essential interventions and to identify areas for development.

**Results:**

Contact indicators from pregnancy to childhood were identified as current indicators for immediate use, but indicators reflecting the quality of care provided during these contacts need development. At each contact point, some essential interventions can be measured now, but the need for development of indicators predominates around interventions at the time of birth and interventions to treat infections. Addressing this need requires improvements in routine facility based data capture, methods for linking provider and community–based data, and improved guidance for effective coverage measurement that reflects the provision of high–quality care.

**Conclusion:**

Coverage indicators for some essential interventions can be measured accurately through household surveys and be used to track progress in maternal, newborn and child health.  Other essential interventions currently rely on contact indicators as proxies for coverage but urgent attention is needed to identify new measurement approaches that directly and reliably measure their effective coverage.

Within the 17 Sustainable Development Goals (SDGs) a total of 169 targets and over 230 indicators have been defined [1]. In alignment with the SDGs, the Global Strategy for Women’s, Children’s and Adolescents’ Health (the Global Strategy) has described an ambitious action and measurement agenda around the three pillars “Survive, Thrive and Transform” [2]. In the immediate future many countries have an unfinished agenda to accelerate the scale–up of cost–effective essential maternal, newborn and child health (MNCH) interventions that save lives as well as help families to thrive [3]. Tracking intervention coverage is a top priority to assist this scale–up so that countries know the extent to which populations in need are benefiting, and delivery strategies are refined as a result [4]. In this paper we examine priorities in coverage measurement of essential MNCH interventions, distinguishing between those that can be measured now and those that require methodological development.

Of particular importance is to explicitly acknowledge known measurement challenges across the continuum from pregnancy to childhood [5–7], and categorise indicators that can be measured now using existing methods and tools (“*indicators for immediate use*”), and those that are high priority in the context of life–saving, quality care but require further methodological development and validation (“*priority indicators for development”*). Once validated using feasible methods, these priority indicators for development can be further described in global guidance and integrated within existing data collection systems.

The remainder of this paper proposes a transparent set of evidence–based considerations for the global MNCH measurement improvement agenda. We draw on evidence supporting cost–effective investments in MNCH [3], recommendations by the Global Strategy [8], and the priorities identified by other initiatives including the Global Reference List of 100 core indicators [9], the World Health Organization’s consultation on quality MNCH [10], the Every Newborn Action Plan (ENAP) [11], and Ending Preventable Maternal Mortality (EPMM) [12].

## METHODS AND CONSIDERATIONS IN SELECTING INDICATORS FOR IMMEDIATE USE

**Figure 1:**
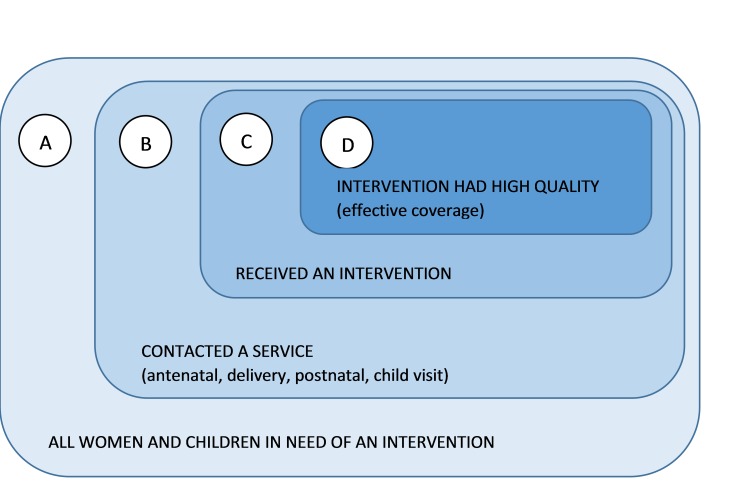
Typology of indicators for maternal, newborn and child health.

**Figure 1** presents a typology of indicators related to intervention coverage. Level A encompasses all women and children who can benefit from receiving care, including preventive and curative services.  From this group, only some will access care and have the opportunity to benefit from the services they need (level B). But making contact with services does not ensure receipt of a specific intervention (level C), irrespective of whether the population making contact needs a preventive or curative intervention. Currently, coverage measurement for any given intervention is defined as C/A, or the proportion of women and children who need an intervention who actually receive it. The innermost element of the framework (level D) highlights the importance of incorporating dimensions of quality within coverage, often referred to as “effective coverage”, for example including measures of appropriate diagnosis, drug dosage, or counselling.  The need for development of globally standardised measures of effective coverage is described in more detail below.

Our considerations for determining the measurement status of indicators builds on the experience and evidence base generated by others, including household survey programs such as Demographic and Health Surveys (DHS) [13] and the Multiple Indicator Cluster Surveys (MICS) [14], the Countdown to 2015 for Maternal, Newborn and Child Survival (Countdown) initiative [15], and the investment and visibility promoted by the Commission on Information and Accountability for Women’s and Children’s Health (CoIA) and its independent Expert Review Group (iERG) [16].

We took five characteristics into account in selecting priority indicators.

1) **Public health importance**. Priority indicators should measure progress in coverage for an intervention that has the potential to save a large number of women’s and children’s lives, because it is linked through known channels to changes in health status. We estimate this potential using the Lives Saved Tool (LiST) [17], calculating the number of maternal, newborn and child lives that could be saved by 2030 based on the underlying assumptions within the model, and if universal coverage was achieved for the intervention in the 75 countries that accounted for 99% of deaths among those groups in 2014, assuming coverage trajectories for all other interventions remain the same (**Table 1**). We have included indicators for malaria and HIV because of their importance in some high burden countries, even though they do not account for large numbers of deaths in all countries.

**Table 1:** LiST analysis of lives saved by labor and delivery management, and life–saving interventions for mothers, newborns and children*

	Estimated number of deaths averted					
**Intervention**	**Stillbirths**	**Neonatal**	**Child**	**Maternal**	**Total**	**Rank**
Labor & delivery management	689 758	549 031		76 850	1 315 639	1
Full supportive care for prematurity		544 458			544 458	2
Full supportive care for sepsis/pneumonia		409 877			409 877	3
Oral Rehydration Solution		12 653	369 423		382 076	4
Water connection in the home			368 313		368 313	5
Treatment with antimalarials			303 653		303 653	6
Oral antibiotics for pneumonia			300 682		300 682	7
Promotion of breastfeeding		74 699	191 976		266 675	8
Hand washing with soap			235 898		235 898	9
Neonatal resuscitation		212 439			212 439	10
Therapeutic feeding for severe wasting			209 442		209 442	11
Injectable antibiotics for neonatal sepsis/pneumonia		181 512			181 512	12
Kangaroo Mother Care		158 853			158 853	13
Syphilis detection and treatment	149 597	7 060			156 657	14
Pneumococcal vaccine			139 779		139 779	15
Improved sanitation			136 256		136 256	16
Clean postnatal practices		131 782			131 782	17
Clean birth practices		101 266		20 148	121 414	18
Treatment for moderate acute nutrition of children			110 671		110 671	19
Immediate assessment and stimulation of newborns		109 585			109 585	20
Hib vaccination			106 998		106 998	21
Zinc–for treatment of diarrhea			106 481		106 481	22
Zinc supplementation			104 426		104 426	23
Magnesium sulphate for pre–eclampsia	64 939			23 681	88 620	24
Homes protected from malaria by ownership of insecticide treated nets or indoor residual spraying			87 733		87 733	25
Chlorhexidine for cord care		82 283			82 283	26
Appropriate complementary feeding			80 081		80 081	27
Intermittent presumptive treatment for malaria in pregnancy	59 942	16 111	1 539	1 404	78 996	28
Oral antibiotics for neonatal sepsis or pneumonia		74 462			74 462	29
Thermal care for newborns		72 391			72 391	30
Hygienic disposal of stools			64 653		64 653	31
Periconceptual Folic Acid / Ferrous Sulfate	17 711	43 296			61 007	32
Antibiotics for premature preterm rupture of membranes		49 257		7 903	57 160	33
Rotavirus vaccine			56 788		56 788	34
Induction of labor for pregnancies beyond 42 weeks	47 230				47 230	35
Balanced energy protein supplementation for pregnant women		41 268	3309		44 577	36
Multiple micronutrients for pregnant women		39 615	2788		42 403	37
Active management of third stage of labor				33 782	33 782	38
Case management of maternal sepsis				23 528	23 528	39
Iron supplementation for pregnant women		21 964	1555		23 519	40
Diabetes case management for pregnant women	22 585				22 585	41
Magnesium sulfate for treatment of eclampsia				22 572	22 572	42
Improved water			21 470		21 470	43
Case management of hypertensive disorders in pregnant women				20 025	20 025	44
Safe abortion services				15 529	15 529	45
DPT3 vaccination			15 428		15 428	46
Tetanus toxoid vaccination		14 940		161	15 101	47
Vitamin A supplementation			14 967		14 967	48
Vitamin A–for treatment of measles			14 574		14 574	49
Post abortion case management				13 391	13 391	50
Calcium supplementation				8124	8124	51
Ectopic pregnancy case management				2980	2980	52
Case management of malaria in pregnant women				2347	2347	53
Antibiotics for dysentery			1017		1017	54

Hib – *Haemophilus influenzae* type B, DPT3 – diphtheria–tetanus–pertussis

*The potential number of lives saved by 54 evidence based interventions by 2030, estimated using the Lives Saved Tool if universal coverage was achieved for each intervention in the 75 countries that accounted for 99% of maternal, newborn and child deaths in 2014, assuming coverage trajectories for all other interventions remain the same.

2) **Feasibility and affordability**. Indicators for immediate use must be affordable and feasible for accurate measurement in the majority of high–MNCH mortality countries to inform immediate actions.  But high–impact interventions for which feasible and cost–effective measurement strategies are not currently available must not be lost and are the target of an urgent developmental research agenda, described below under *priority indicators for development*.

3) **Accuracy.** Measurement approaches that do not produce valid results are a waste of scarce resources, and can misdirect policy and program decisions. There is a growing body of research demonstrating that mothers interviewed during household surveys (as in DHS or MICS) can report accurately on whether they and their children received some interventions, but not others.  Particularly problematic are high impact interventions around the time of birth and curative interventions for episodes of illness such as antibiotics for pneumonia [18–20]. New and innovative approaches for measuring coverage for these interventions are needed urgently, while maintaining support for household surveys able to produce highly–accurate estimates of coverage for most MNCH interventions. Surveys are also essential for assessing equity through disaggregated analyses, as required by SDG target 17.18 on the measurement of inequalities.

4) **Production of timely results with clear action implications.** Indicator levels should change in response to increases or decreases in program inputs and outputs and improvements in program processes, within a time frame of one to three years, to provide information useful to program managers. Experience has demonstrated that monitoring systems work best and are more likely to be sustained if the data they contain are used first at the level at which they are collected, and also at each higher level throughout the reporting system. Of importance is to encourage reporting and use of individual indicator components from the point of data collection through national level, but combining the components for global monitoring.

5) **Consistency with historical indicators, to permit tracking of trends.** Lists of indicators evolve over time. New interventions are scaled up that require new indicators, but also the validity of existing indicators may be challenged by new evidence. For example, the indicator for diarrhea management used in most surveys since the 1990s was oral rehydration therapy (ORT), but more recently there has been a shift towards reporting on oral rehydration salts (ORS) plus zinc [21]. For the purpose of assessing time trends as we transition from the Millennium Development Goals (MDGs) to the SDGs it is useful to continue to report on ORT as well as ORS for a period of time, while also designing measurement methods so that adjustments to indicator definition can be made.

## GETTING STARTED: INDICATORS FOR IMMEDIATE USE

**Figure 2:**
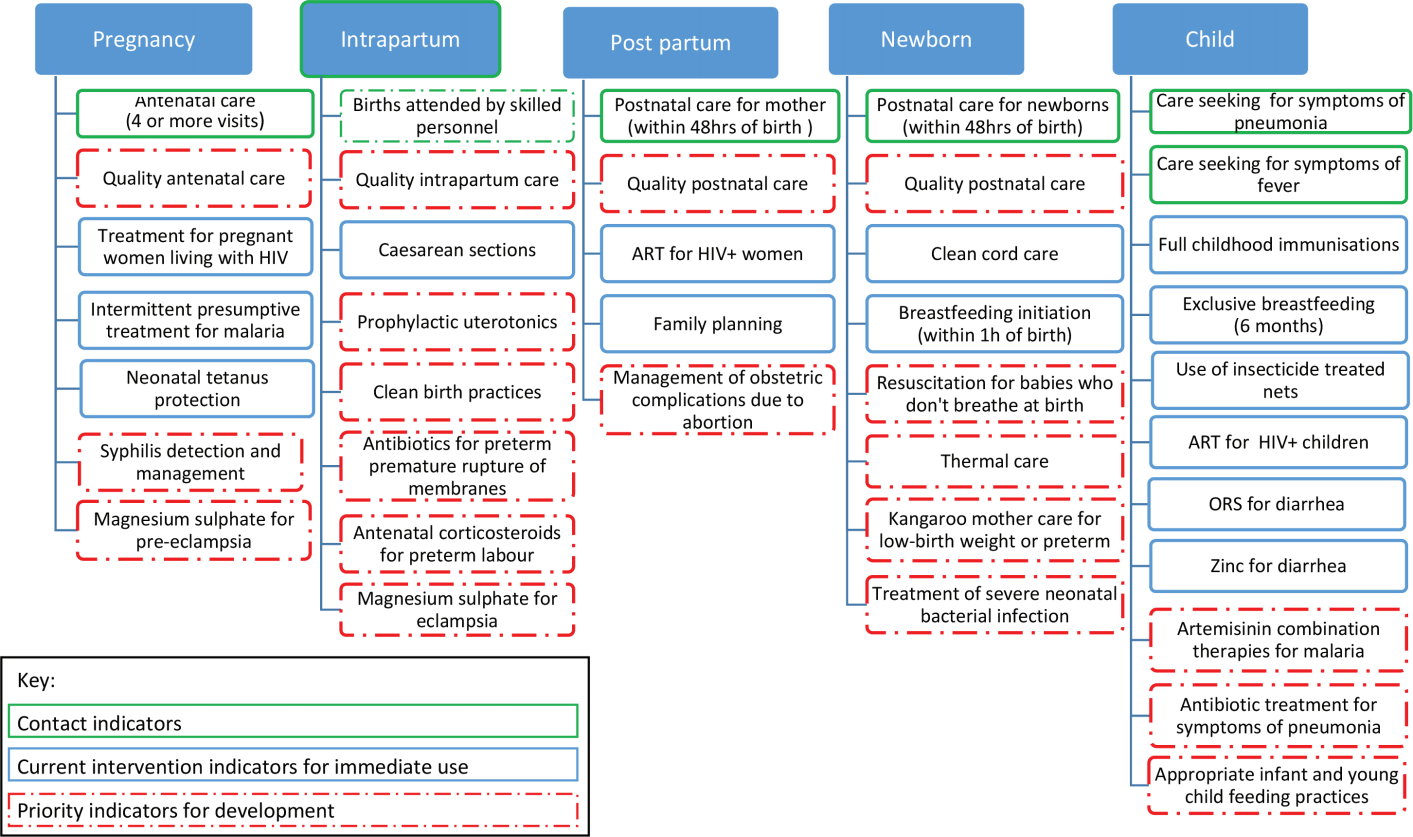
Measurement status of priority contacts and evidence based interventions across the continuum from pregnancy to childhood.

In **Figure 2** we present the contacts and interventions prioritized by different global groups in MNCH (for example ENAP, EPMM, the Global Strategy), and include those supported by evidence of impact from LiST analysis (**Table 1**). After consideration of the five characteristics above these have been categorized as “*current*” or “*priority for development*”.

Contacts are included in order to measure the proportion of individuals accessing care, and thus potential to receive interventions, corresponding to level B in **Figure 1**. In addition to the contacts for antenatal care, skilled attendant at birth, and postnatal care, we also include care seeking for sick children (specifically fever and symptoms of childhood pneumonia), consistent with the typology that distinguishes accessing care from actual receipt of a life–saving intervention. Correct treatment of these two conditions are among the highest–impact interventions, but cannot be measured accurately through household surveys. We also indicate the need to develop, agree on and validate indicators that reflect quality care at these contact points to enable tracking of effective coverage measures [22]. The remainder of **Figure 2** presents intervention indicators. High impact interventions are represented across the continuum from pregnancy to childhood and measurement development needs are identified at each stage. Addressing these needs requires immediate action, as described in the next section.

## DOING BETTER: AN ACTION AGENDA FOR IMPROVED MEASUREMENT

Priority indicators for development predominate around interventions at the time of birth, interventions to treat infections, and quality of care. Some of these represent relatively rare events (for example antibiotics for preterm premature rupture of membranes) and may never be suitable for population level tracking at national level, but nonetheless require advances in measurement in order to report accurately to country programs. For many, service contact indicators have been used to represent imperfect proxy measures of care but the need for measures of quality care means that we have to do better.   For example, the service contact indicator “skilled attendant at birth” is the most widely used proxy indicator for care at birth, but the evidence linking increases in skilled attendant coverage with reductions in mortality has not been consistent [23–25], probably reflecting the fact that only a subset of locally–defined skilled attendants actually have the skills, commodities and facilities needed to deliver essential interventions at birth.

We propose that four specific types of measurement innovations are required.

First, a measurement improvement agenda is needed for routine data capture, so that the accuracy of reporting clinical interventions for women, newborns and children is improved at different levels of the health system. This will allow delivery of high impact interventions to be tracked at local, national and global levels. It will require improved routine data systems, review and consolidation of facility assessment tools and methods, and engagement with health system strengthening efforts more broadly.

Second, to realize the potential of these improved data sources, methods for linking population and provider–based data sources are needed [5,17]. Household survey methods provide population level data and permit equity analysis but can be limited by poor recall and infrequent reporting. Facility data can be continuous and timely, has potential to improve reporting on clinical events, and can be stratified by level and type. However, present reporting tools cannot provide accurate equity breakdowns or population level estimates. Combining these two data streams has the potential to be transformative for monitoring the delivery of essential interventions that cannot currently be measured reliably, and for measuring effective coverage so that coverage indicators are defined as level D/A in **Figure 1**.

Third, further advances in implementation science are needed in order to place indicator development in the context of research on the design, implementation and impact of large scale programs.

And fourth, as new measures and approaches are tested and proven ready for wider adoption, global resources and guidance should be developed. Resources would include access to questionnaires, forms, and protocols; perhaps in one accessible system. Global guidance would include definitions, strengths and limitations of potential data sources, and interpretation notes.

## TAKING THE AGENDA FORWARD

This paper adds to other recent calls for improved measurement that can enhance accountability and refine strategies to save lives [26]. At this time of transition from the MDGs to the SDGs it is essential that baselines are established, ambition is maintained, guidance and resources are shared, and momentum is not lost. Clarity about which essential interventions can be measured directly, reliably and feasibly using existing methods is an integral part of that plan. But here we also identify the need for focused, intensive commitment to advance the coverage measurement agenda for all essential interventions–especially those that save lives during and immediately after childbirth, and for sick children–so that we progress from reliance on measuring contacts with health care providers to measuring the effective coverage of clinical high–impact interventions.

As we enter the SDG era, several key partners are stepping forward to join this global measurement agenda for maternal, newborn and child health to agree on priorities, to coordinate actions and learning, and to work together with countries so that ownership of and capacity for an improved measurement agenda sits where the ability to act on evidence is greatest.
